# Impaired autophagy-accelerated senescence of alveolar type II epithelial cells drives pulmonary fibrosis induced by single-walled carbon nanotubes

**DOI:** 10.1186/s12951-023-01821-6

**Published:** 2023-02-28

**Authors:** Xiang Zhang, Xinxin Hu, Yuqing Zhang, Bin Liu, Haihong Pan, Zikai Liu, Zhuomeng Yao, Qixing Zhu, Changhao Wu, Tong Shen

**Affiliations:** 1 Department of Occupational Health and Environment Health, School of Public Health, Anhui Medical University, Hefei, 230032 China; 2 Department of Medical Aspects of Specific Environments, School of Basic Medicine, Anhui Medical University, Hefei, China; 3 Department of Biochemistry and Physiology, Faculty of Heath and Medical Sciences, University of Surrey, Surrey, Guildford UK

**Keywords:** Carbon nanotube, Alveolar type II epithelial cells, Autophagy, Senescence, Pulmonary fibrosis

## Abstract

**Background:**

The rapid increase in production and application of carbon nanotubes (CNTs) has led to wide public concerns in their potential risks to human health. Single-walled CNTs (SWCNTs), as an extensively applied type of CNTs, have shown strong capacity to induce pulmonary fibrosis in animal models, however, the intrinsic mechanisms remain uncertain.

**Results:**

In vivo experiments, we showed that accelerated senescence of alveolar type II epithelial cells (AECIIs) was associated with pulmonary fibrosis in SWCNTs-exposed mice, as well as SWCNTs-induced fibrotic lungs exhibited impaired autophagic flux in AECIIs in a time dependent manner. In vitro, SWCNTs exposure resulted in profound dysfunctions of MLE-12 cells, characterized by impaired autophagic flux and accelerated cellular senescence. Furthermore, the conditioned medium from SWCNTs-exposed MLE-12 cells promoted fibroblast-myofibroblast transdifferentiation (FMT). Additionally, restoration of autophagy flux with rapamycin significantly alleviated SWCNTs-triggered senescence and subsequent FMT whereas inhibiting autophagy using 3-MA aggravated SWCNTs-triggered senescence in MLE-12 cells and FMT.

**Conclusion:**

SWCNTs trigger senescence of AECIIs by impairing autophagic flux mediated pulmonary fibrosis. The findings raise the possibility of senescence-related cytokines as potential biomarkers for the hazard of CNTs exposure and regulating autophagy as an appealing target to halt CNTs-induced development of pulmonary fibrosis.

**Graphical Abstract:**

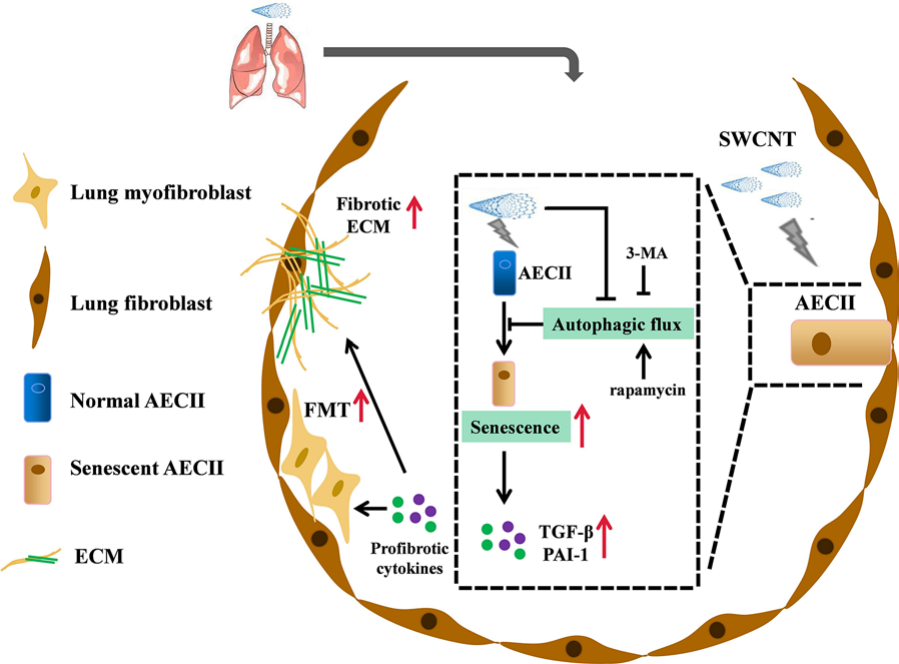

**Supplementary Information:**

The online version contains supplementary material available at 10.1186/s12951-023-01821-6.

## Introduction

Carbon nanotubes (CNTs), as one major class of engineered nanomaterials, were widely used in numerous fields [1]. The rapidly growing production and application of CNTs affect millions of workers and users. Several studies reported that the concentration of CNTs exceeded recommended exposure limit (REL) in workplace [2, 3]. In addition, one study analyzed internal exposure to CNTs via sputum analysis and found that 18% of participants had CNT present in the sputum [4]. Whole-body inhalation to CNTs has been confirmed to result in CNT deposition in the lungs and obvious lung injure based on animals models [5, 6]. Therefore, the lung is the vital target organ of CNTs exposure, and concerns regarding inhalation toxicity of CNTs exposure continue to mount in recent years [7, 8]. Single-walled CNT (SWCNT) is an extensively applied type of CNTs and present in stiff, rope-like bundles with extremely small diameter and high surface area. Existing evidence suggests that SWCNTs exposure can give rise to the respiratory toxicity, such as lung inflammation and pulmonary fibrosis [9]. Our previous study reported that SWCNTs showed stronger capacity to induce pulmonary fibrosis compared to multi-walled CNTs (MWCNTs) [10, 11]. Therefore, research into the mechanisms by which SWCNTs induce pulmonary fibrosis is warranted in order to facilitate the understanding, monitoring, prevention and treatment of SWCNTs-induced lung lesions that might occur in exposed populations.

Pulmonary fibrosis is a form of chronic lung disease caused by aberrant repair response to constant alveolar epithelial cells (AECs) injury and fibroblast-myofibroblast transdifferentiation (FMT) [12, 13]. Alveolar type II epithelial cells (AECIIs), as progenitors of the alveolar epithelium, play an essential role in maintaining homeostasis of alveolar structure and function [14, 15]. One study reported that cellular senescence was essential for the pathophysiological progression of pulmonary fibrosis induced by bleomycin [16]. Recent studies have revealed that virus-induced senescence of AECIIs may cause pulmonary fibrosis following coronavirus disease 2019 (COVID-19) infection [17, 18]. These findings suggest that the senescence of AECIIs is the primary driver for the progression of pulmonary fibrosis. Senescent AECIIs fail to regenerate AECIs lost by injury and do not respond normally to epithelial damage, undergoing hyperplasia in pro-fibrotic conditions [19]. Furthermore, senescent AECIIs could produce senescence-associated secretory phenotype (SASP) molecule, which trigger FMT and persistent lung tissue remodeling [20–23]. Preventing senescence of AECIIs or targeting senescent cells can reduce the risk of pulmonary fibrosis [24]. To date, there has been limited advance on the pathological mechanisms underlying the senescence of AECIIs and specific effects of targeting senescent AECIIs on pulmonary fibrosis induced by SWCNTs.

Autophagy is a homeostatic process that recycles intracellular materials in response to various cellular stresses, which could inhibit senescence-related inflammation, maintain cellular and tissue homeostasis and the regenerative capacity of stem cells [25, 26]. Impaired autophagy is one of the features of cellular senescence, and inflammation caused by autophagy impairment is a major factor of senescence-induced tissue damage [27, 28]. Defective autophagy of AECIIs has been reported to cause its senescence and damage, resulting to abnormal epithelial-mesenchymal transition (EMT) and promoting FMT during the idiopathic pulmonary fibrosis. Meanwhile, restoring autophagy inhibited TGF-β and reduced collagen deposition [29, 30]. Therefore, boosting autophagy to halt senescence and regulating pulmonary fibrosis process through the disruption of specific molecular pathways is a promising therapeutic avenue. Existing evidence shows that exposure to MWCNTs inhibits autophagic flux in murine macrophages [31]. However, it remains elusive so far whether impairing autophagic flux-mediated senescence of AECIIs contribute to SWCNTs-induced pulmonary fibrosis. This study was to clarify the regulatory relationship between impairment of autophagic flux and senescence of AECIIs after SWCNTs exposure in vivo and in vitro. Our findings bring novel insights into the role of autophagy regulating AECIIs senescence during SWCNTs-induced pulmonary fibrosis, and add to the current awareness of AECIIs as an important player in the pulmonary disease.

## Methods

### Reagents

SWCNTs (XFS05) were purchased from Nanjing XFNANO Materials Tech Co., Ltd., 3-(4,5)-Dimethylthiahiazo(-z-y1)-3,5-di-phenytetrazoliumromide (MTT) and rapamycin (V900930) were purchased from Sigma-Aldrich (St. Louis, USA). Dulbecco's modified Eagle’s medium (DMEM) and foetal bovine serum (FBS) were purchased from HyClone (Logan, USA), and goat serum was purchased from Boster Biological Technology Co. Ltd. (Wuhan, China). 3-MA was from (19312) MedChem Express (New Jersey, USA). Rabbit monoclonal anti-α-SMA (ab32575) and mouse monoclonal anti-β-actin (ab20272) were purchased from Abcam (Cambridge, MA). Rabbit monoclonal anti-p21 (64016S), rabbit monoclonal anti-autophagy related 5 (Atg5) (12994S), rabbit monoclonal anti-p62 (39749) and mouse monoclonal anti-LC3B (83506) were purchased from Cell Signaling Technology (Beverley, MA). Mouse monoclonal anti-COL I (sc-59772) and mouse monoclonal anti-p16 (sc-1661) were purchased from Santa Cruz (California, USA). Rabbit polyclonal anti-SP-C (DF6647) was purchased from Affinity Biosciences (Cincinnati, USA). ELISA kits were obtained from Calvin Biotechnology Co., Ltd. (Suzhou, China). Haematoxylin, eosin, Masson’s trichrome and senescence-associated-β-galactosidase (SA-β-Gal) staining kits were purchased from Solarbio Science & Technology (Beijing, China).

### Animal experiments

Eight-week-old female C57BL/6J mice were obtained from the Laboratory Animal Centre of Anhui Medical University. All mice were maintained in a standard environment for 1 week. To examine the effect of SWCNTs on autophagy and cellular senescence in AECIIs at different stages, the mice were administered a single dose of 40 μg SWCNTs in 50 μl of PBS by intratracheal instillation, and mice in the control (CTRL) group were treated with 50 μl PBS. This dose has been shown to be relevant to human exposures at workplace [32]. The characteristics of SWCNTs were described in our previous study [10]. Briefly, the length of SWCNT was 1–3 µm with the diameter of 1–2 nm. Highly negative zeta-potential showed that SWCNTs were relatively well dispersed in PBS. The endotoxin in SWCNTs suspension was below 0.1 EU/ml. Bronchoalveolar lavage fluid (BALF) collection and lung tissue isolation were conducted as previously described on days 3, 7 and 28 after SWCNTs administration [10]. All procedures performed on mice followed the guidelines for humane treatment by the Association of Laboratory Animal Sciences at Anhui Medical University.

### Haematoxylin and eosin (HE) staining and Masson staining

Paraffin-embedded lung tissues were subjected to HE and Masson’s trichrome staining to observe morphological alterations and fibrotic changes, respectively. HE and Masson staining were carried out according to a standard protocol, and slides of lung tissues were observed by light microscopy (ZEISS, Germany). Each successive field (40 fields each sample) was individually assessed for the severity of pulmonary fibrosis in a blinded fashion using the Ashcroft scoring system [33].

### Hydroxyproline (HYP) assay

Fifty milligrams of lung tissue were homogenized and hydrolyzed using 6 mol/l hydrochloric acid for 6 h at 100 °C. Then, the hydrolysate was neutralized with 10 mol/l sodium hydroxide. HYP levels in the hydrolysate were measured by HYP kits according to the manufacturer’s instructions. The absorbance was measured at 560 nm, and the level of HYP in lung tissues was calculated.

### Cell culture

The mouse AECIIs line MLE-12 cells were obtained from Shanghai Fuheng Biotechnology Co., Ltd. and cultured in DMEM containing 10% FBS in 5% CO_2_ at 37 °C. Primary lung fibroblasts were isolated from 4-week-old C57BL/6J mice according to our earlier study [10]. Briefly, the pulmonary left lobe of mice was fully cut into 1 mm^3^ pieces and digested with 0.5 mg/ml trypsin, then filtered through nylon mesh. The pellet was resuspended using DMEM with 15% FBS and cultured under 5% CO_2_ at 37 °C. The adherent cells were as fibroblasts and harvested for passage or other assays.

### MTT assay

The modified MTT assay, as previously described, was to determine the cytotoxicity of SWCNTs in vitro [34]. MLE-12 cells were seeded in 96-well plates and treated with different doses of SWCNTs (0, 5, 10, 25, 50 µg/ml) for 6, 12, 24 and 48 h. Then, 10 µl of MTT solution (5 mg/ml) was added and incubated for 4 h at 37 °C. After being incubated with MTT solution, different doses of SWCNTs were added into the control medium as SWCNTs interference (SI group) to measure the absorbance of SWCNTs causing “false” values of cytotoxicity. Then contribution of such values was taken into account to obtain the real data. Dimethyl sulfoxide was added to the wells and the OD at 570 nm was measured. MLE-12 cell viability is shown as the ratio of OD_SWCNT_ to OD_SI_.

### Cell treatment

To examine the time effect of SWCNTs on autophagy and senescence in AECIIs and subsequent FMT in primary lung fibroblasts, MLE-12 cells were treated with SWCNTs (10 µg/ml) for different time points (0, 6, 12 and 24 h). The culture medium of MLE-12 cells was centrifuged at 12,000 r for 10 min at 4 °C to precipitate cells and CNTs, then the supernatants of culture medium from each group were collected and used as conditioned medium (CM) to culture with primary mouse lung fibroblasts for 48 h. To examine the effect of SWCNTs-impaired autophagic flux on senescence and subsequent FMT, MLE-12 cells were pretreated with 500 nM rapamycin (an autophagy inducer) or 5 mM 3-MA (an autophagy inhibition) for 24 h before SWCNT (10 µg/ml) exposure for 24 h, and then CM experiments were conducted.

### Western blotting

Lung tissue of mice, MLE-12 cells and primary lung fibroblasts were homogenized in RIPA lysis buffer, and the protein concentration was determined by the BCA method. Total proteins were adjusted to 2 µg/µl, separated by 10–12.5% SDS–PAGE and transferred to polyvinylidene fluoride membranes. The membranes were blocked in 5% fat-free powdered milk in TBST at room temperature for 2 h and then incubated with anti-p16 (1:1000), anti-Atg5 (1:1000), anti-p21 (1:1000), anti-LC3B (1:1000), anti-p62 (1:750), anti-α-SMA (1:2000) and anti-β-actin (1:2000) antibodies at 4 °C overnight. Following primary antibody incubation, the membranes were washed and then incubated with goat anti-rabbit (mouse) IgG (1:10,000) for 2 h at room temperature. An enhanced chemiluminescence detection kit was used to visualize the signals. β-actin was used as the loading control. Images were analyzed by *Image J* software.

### Immunohistochemistry (IHC)

Sections of paraffin-embedded lung tissues were deparaffinized and then treated with 3% hydrogen peroxide for 30 min to block endogenous peroxidase activity. After being washed three times with PBS, antigen retrieval was performed. Next, the sections were stained with anti-LC3B (1:200) at 4 °C overnight. Then, the sections were washed and incubated with secondary antibodies for 30 min at 37 °C, followed by SP, the DAB reaction and haematoxylin staining. Sections were observed by light microscopy (ZEISS, Germany). Cells with brownish yellow particles were considered to have positive expression.

Cultured primary lung fibroblasts were fixed with 4% paraformaldehyde for 10 min and then permeabilized with 0.1% Triton X-100 for 15 min. Endogenous peroxidase was blocked using 3% hydrogen peroxide. Cells were incubated with goat serum and then stained with anti-COL I antibodies (1:200) at 4 °C overnight. Then, the cells were washed using PBS and incubated with secondary antibodies for 30 min at 37 °C, followed by SP, the DAB reaction and haematoxylin staining. Cells were observed by light microscopy (Olympus, Tokyo, Japan).

### Immunofluorescence (IF) analysis

Cryosections of mouse lung tissues were washed and incubated with goat serum to block nonspecific binding sites. Next, the cryosections were incubated with anti-SP-C antibodies (1:200) and anti-LC3B antibodies (1:200) or anti-SP-C antibodies (1:200) and anti-p16 antibodies (1:200) at 4 °C overnight. The cryosections were washed and incubated with FITC- or AF594-conjugated secondary antibodies (Elabscience) in the dark. Nuclei were labelled with DAPI (BL105A, Biosharp). Images were acquired using a fluorescence microscope (Olympus, Japan).

Fixed MLE-12 cells were permeabilized using 0.1% Triton X-100 for 15 min, blocked using goat serum for 30 min and incubated with anti-LC3B antibodies (1:200). Then, the cells were incubated with AF594-conjugated secondary antibodies (E-AB-1060, Elabscience) in the dark at 37 °C for 30 min. The nuclei were counterstained with DAPI (BL105A, Biosharp). Images were acquired using a fluorescence microscope (Olympus, Japan).

### SA-β-Gal staining

SA-β-Gal activity was examined using a SA-β-Gal staining kit according to the manufacturer’s instructions. Briefly, fixed MLE-12 cells were incubated with SA-β-gal staining solution containing 1 mg/ml X-Gal overnight. Senescent cells were identified as bluish green-stained cells under a light microscope (Olympus, Tokyo, Japan).

### ELISA

Mouse BALF and culture medium were collected and centrifuged at 12,000 r for 15 min at 4 °C to precipitate the cells and SWCNTs to exclude the possible influence of cells and SWCNTs on ELISA results, then the supernatants of BALF and culture medium were obtained. The levels of SASP factors (TGF-β and PAI-1) were quantified by ELISA kits according to the instructions.

### Real-time quantitative polymerase chain reaction (RT–qPCR)

A Transcription First Strand cDNA Synthesis kit (Roche Diagnostics GmbH, Germany) was used to generate cDNA from mRNA isolated from cells using TRIzol reagent. RT–qPCR was performed with Light Cycler 480 SYBR Green I Master Mix (Roche Diagnostics GmbH, Germany), and gene-specific primers are shown in Additional file 1: Table S1. The PCR amplification reactions were as follows: 95 °C for 5 min and 45 cycles of a three-step PCR (95 °C for 15 s, 60 °C for 15 s and 72 °C for 30 s). The amount of the target was calculated using the standard 2^−ΔΔCt^ method with β-actin serving as an endogenous reference.

### Statistical analysis

The data are presented as the *mean* ± *SEM*. Statistical analysis was performed using *SPSS 23.0* software (SPSS Inc., Chicago, IL). The difference between control and treated samples was examined by t-test. For multiple group comparisons, one-way analysis of variance with a post hoc test using the LSD method was conducted. *P* < 0.05 was considered significant. All data are representative of at least three different experiments.

## Results

### Accelerated senescence of AECIIs during pulmonary fibrosis induced by SWCNTs

**Fig. 1:**
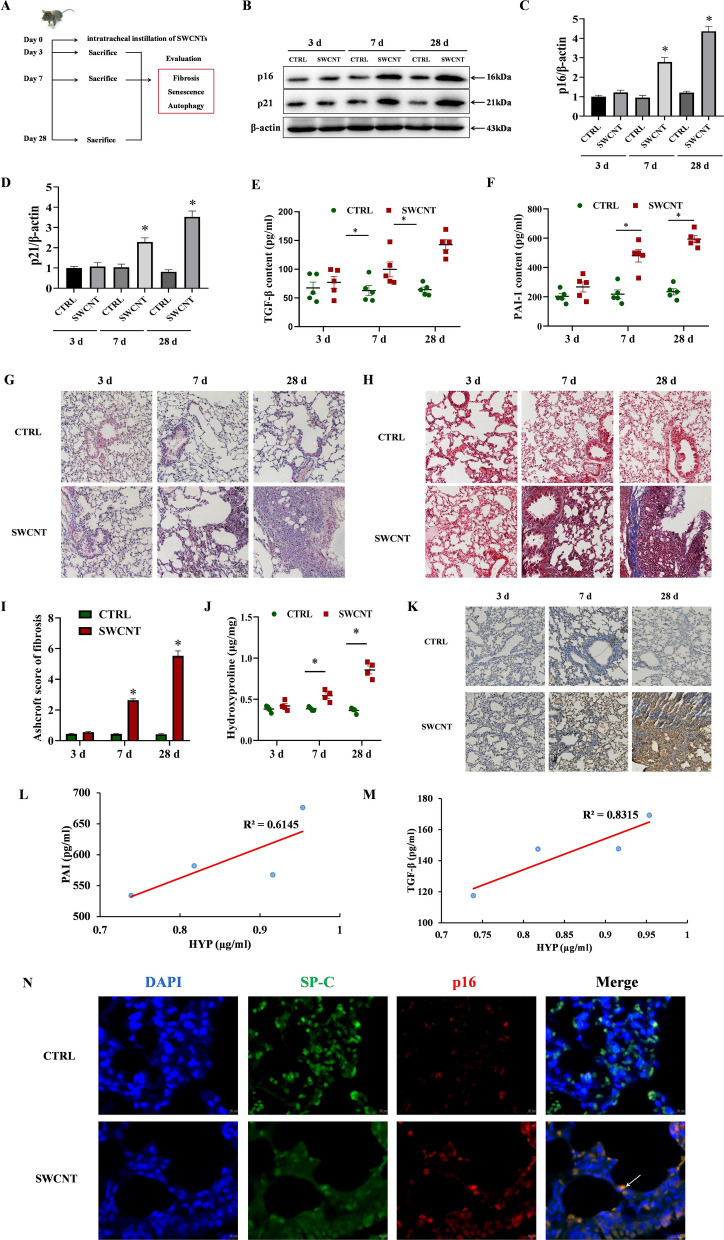
Single-walled carbon nanotubes (SWCNTs) induced pulmonary fibrosis and senescence of AECIIs in mouse lung tissues. Mice were exposed to 40 μg SWCNT by intratracheal instillation, then lung tissues were collected on days 3, 7 and 28. **A** Scheme of workflow for evaluation of cellular senescence and pulmonary fibrosis induced by SWCNTs in vivo. **B**–**D** Western Blotting analysis of p21 and p16 protein expressions (n = 3) in lung tissues. Contents of TGF-β (**E**) and PAI-1 (**F**) in bronchoalveolar lavage fluid (BALF) quantified by ELISA (n = 5). HE staining (**G**) and Masson’s trichrome staining (**H**) of mouse lung tissues (200×). **I** The semiquantitative Ashcroft scores for the severity of pulmonary fibrosis (n = 4). **J** The hydroxyproline (HYP) level (n = 4) in lung tissues of mice. **K** Immunohistochemistry (IHC) of COL I expression (n = 5) in lung tissues of mice. **L**, **M** The correlation of hydroxyproline contents in mice lung tissues and senescence-associated secretory phenotype (SASP) factors (TGF-β and PAI-1) in BALF. **N** Immunostaining for p16 (a senescence-related marker) and SP-C (an AECIIs marker) from SWCNTs-exposed lung tissues of mice on day 28 was detected by immunofluorescence (IF) (400×). *P < 0.05 vs CTRL group

In order to determine whether cellular senescence is accelerated during pulmonary fibrosis induced by SWCNTs, we firstly examined the expressions of p21, p16 and SASP (TGF-β and PAI-1) in lung tissues from mice challenged with SWCNTs exposure after days 3, 7 and 28 (Fig. 1A). The protein levels of p16 and p21 in lung tissue were progressively increased in SWCNTs-treated mice on days 7 and 28 after instillation compared with those in PBS-treated mice (Fig. 1B–D). The contents of TGF-β and PAI-1 in BALF, reflecting the underlying pathologic changes in the lung tissues, were significantly elevated in SWCNTs exposed mice compared to those in PBS-treated mice on days 7 and 28. The induction of SASP was most prominent on day 28 post-exposure (Fig. 1E, F). These data suggest accelerated cellular senescence is induced in SWCNTs-exposed lungs.

Next, we detected pathological alterations of lung tissue and the severity of pulmonary fibrosis. As shown by the results of HE and Masson’s trichrome staining of lung sections, PBS-instilled mice exhibited no fibrotic burden (Fig. 1G–I). In contrast, mice in SWCNTs-instilled groups developed significantly exacerbated pulmonary fibrosis, as evidenced by the progressive thickening of alveolar walls, destruction of alveoli structure and augmented collagen deposition, compared with the control mice on days 7 and 28 after SWCNTs exposure (Fig. 1G–I). Ashcroft scores on histology in lungs and the contents of HYP were also elevated in the lungs of SWCNTs-exposed mice (Fig. 1I, J). Additionally, IHC showed increased expression of COL I, a marker reflecting the accumulation and activation of myofibroblasts, in lung of SWCNTs-exposed mice in a time-dependent manner (Fig. 1K). We further evaluated the correlation between cellular senescence and pulmonary fibrosis induced by SWCNTs. As shown in Fig. 1L, M, there was a positive correlation between SASP (TGF-β and PAI-1) and pulmonary fibrosis under conditions of SWCNTs-exposed lungs of mice (*r* = 0.91 and 0.78).

In order to definite the type of senescent cell, we next performed double immunostaining for p16 (a senescence-related marker) and SP-C (an AECIIs marker). The results illustrated that AECIIs harbored the high p16 expression in SWCNT-exposed lung on day 28 (Fig. 1N). Taken together, these data demonstrate that SWCNTs induce accelerated senescence of AECIIs in mouse fibrotic lungs.

### Impaired autophagic flux in AECIIs from SWCNTs exposed mice

**Fig. 2:**
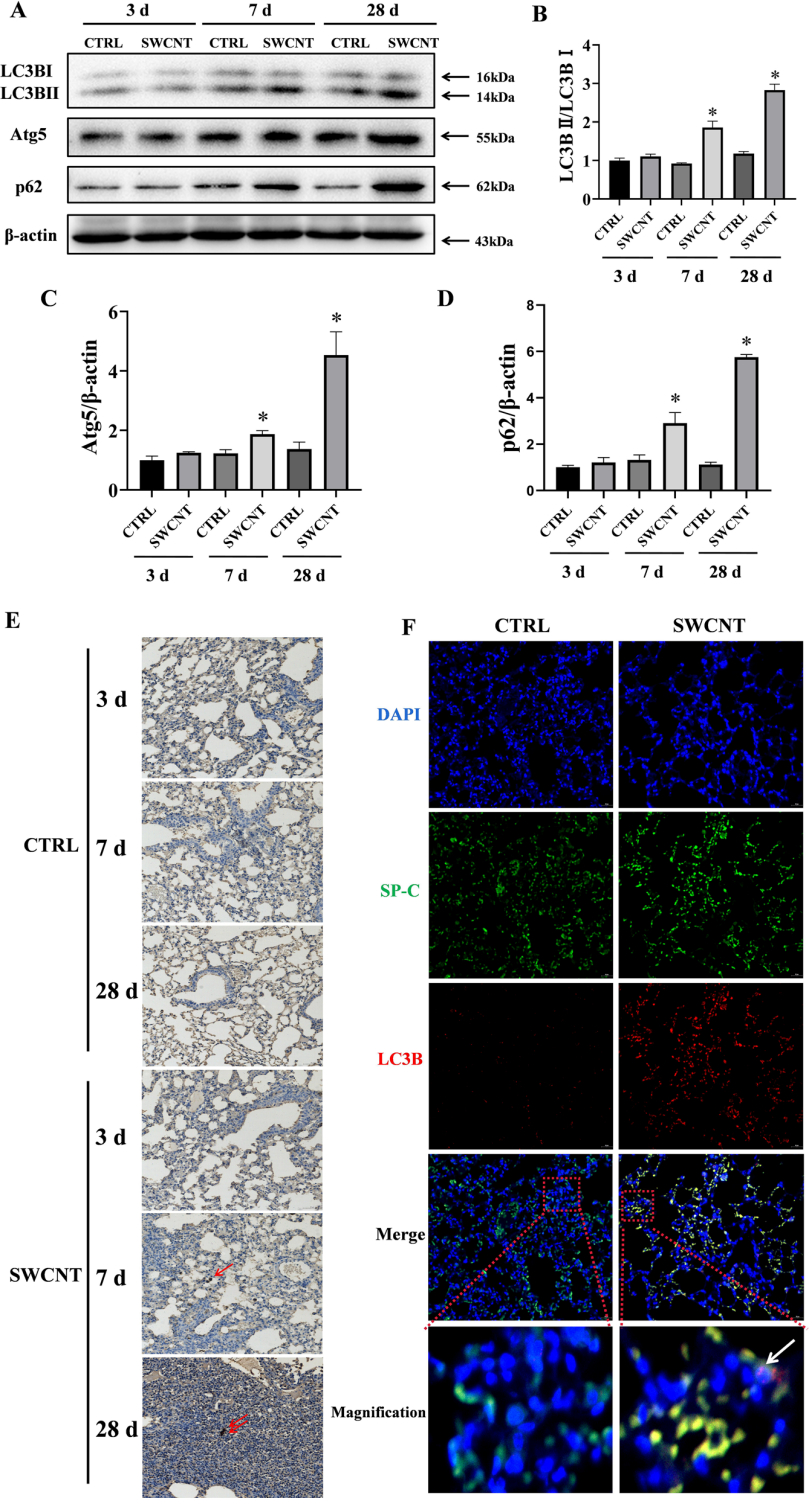
SWCNTs impaired autophagic flux in AECIIs of mouse lung tissue. Mice were exposed to 40 μg SWCNTs by intratracheal instillation, then autophagy of the lung tissue was evaluated. **A**–**D** Western Blotting analysis of Atg5, LC3BI, LC3BII and p62 protein expressions (n = 3) in lung tissues of mice. **E** The LC3B expressions (n = 5) in lung tissues were evaluated by IHC on days 3, 7 and 28 upon SWCNTs administration (200×). **F** Immunostaining for LC3B and SP-C from SWCNTs-exposed lung tissues of mice on day 28 was detected by IF (200×). *P < 0.05 vs CTRL group

The induction of cellular senescence caused by SWCNTs might be related to an accompanying dysfunction of autophagic flux because the latter has been proposed to contribute to pulmonary fibrosis by inducing AECIIs dysfunction [29]. To address this question, we evaluated the change of autophagy in PBS- and SWCNT-exposed lungs of mice. The lipidation of LC3 and its association with autophagosome membranes has been established as a useful sign of autophagy, and Atg5 is a key component of autophagy, enhancing the lipidation of LC3-I to LC3-II. Another widely used marker of autophagy degradation is the autophagy receptor sequestosome 1 (SQSTM1, p62), which physically links autophagic cargo to the autophagic membrane and reflects autophagic flux. We examined Atg5, LC3B and p62 expressions in the lung tissues of mice. No significant modifications in the Atg5, LC3B-II/LC3B-I ratio and p62 protein expressions were observed on day 3 after SWCNTs administration. Importantly, a significant increase in the LC3B-II/LC3B-I ratio, Atg5 and p62 protein expressions was shown in SWCNT-exposed lung tissues on days 7 and 28, indicating the impaired autophagic flux in mice lung tissues response to SWCNTs (Fig. 2A–D). Moreover, LC3B puncta were clearly visible by IHC in the lung tissue of SWCNTs-treated mice on days 7 and 28, whereas diffuse staining was visualized in the CTRL group (Fig. 2E). Because of the crucial role of autophagy in AECIIs in the development of pulmonary fibrosis, we further examined autophagy-related protein in AECIIs. We performed immunostaining for LC3B and SP-C (an AECIIs marker). The results showed a significant increase in LC3B puncta in AECIIs on day 28 after SWCNTs exposure (Fig. 2F). These results collectively indicate that SWCNTs lead to impaired autophagic flux of AECIIs during the pathogenesis of pulmonary fibrosis.

### Senescence caused by impairing autophagic flux in MLE-12 cells mediated FMT induced by SWCNTs

In vivo experiments revealed that impairing autophagic flux and senescence mainly occurred in AECIIs during SWCNTs-induced pulmonary fibrosis. Therefore, MLE-12 cells, one type of AECIIs cell lines, were selected to explore the regulatory relationship between impairing autophagic flux and senescence induced by SWCNTs in subsequent *vitro* experiments.

**Fig. 3:**
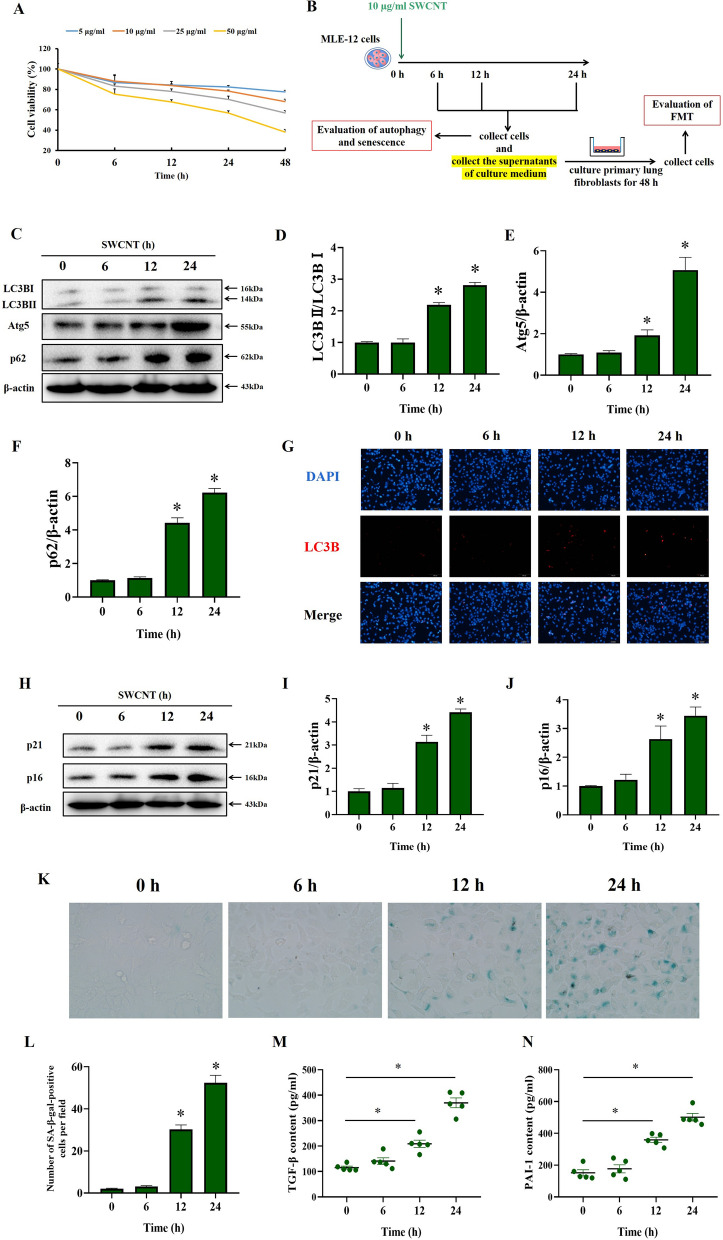
SWCNTs impaired autophagic flux and triggered senescence of MLE-12 cells in vitro. **A** The cytotoxicity of SWCNTs (0, 5, 10, 25 and 50 μg/ml) at different time points (0, 6, 12, 24 and 48 h) was determined by the modified MTT assay. MLE-12 cells were treated with 10 μg/ml SWCNTs for 6, 12 and 24 h, and then the effects of SWCNTs on autophagy and senescence in MLE-12 cells were evaluated. **B** Scheme of workflow for time effects of SWCNTs on autophagy and senescence in MLE-12 cells and fibroblast-to-myofibroblast transdifferentiation (FMT) in lung fibroblasts in vitro. **C**–**F** Western Blotting analysis of Atg5, LC3BI, LC3BII and p62 protein expressions in MLE-12 cells (n = 3). **G** LC3B puncta in MLE-12 cells was observed by IF (200×). **H**–**J** Western Blotting analysis of p21 and p16 protein expressions in MLE-12 cells (n = 3). **K** SA-β-gal activity was determined by X-gal staining in MLE-12 cells (400×). **L** The number of SA-β-gal positive MLE-12 cells per field (n = 8). Contents of TGF-β (**M**) and PAI-1 (**N**) in the supernatants of culture medium quantified by ELISA (n = 5). *P < 0.05 vs CTRL group

In vitro, we first investigated the cytotoxicity of SWCNTs by the modified MTT assay to determine the dose and time points for use in this study. As shown in Fig. 3A, up to 10 μg/ml SWCNTs was not cytotoxic to MLE-12 cells at 6, 12, and 24 h after administration. However, 50 µg/ml SWCNTs showed time-dependent cytotoxicity. Based on the results of the MTT assay, we selected 10 μg/ml as the dose of SWCNTs for use in subsequent experiments.

Next, we determined the effect of SWCNTs exposure on autophagy in MLE-12 cells at various time points (Fig. 3B). As shown in Fig. 3C–F, SWCNTs upregulated the Atg5, LC3B-II/LC3B-I ratio and p62 protein levels in a time-dependent manner. Similarly, a significant increase in LC3B puncta was observed in SWCNT-treated MLE-12 cells (Fig. 3G). These results indicate that SWCNTs exposure causes substantially impaired autophagic process in AECIIs in vitro.

Then, we examined the effect of SWCNTs exposure on senescence of MLE-12 cells in vitro. SWCNTs exposure for 12 and 24 h upregulated the protein expressions of p21 and p16 in MLE-12 cells (Fig. 3H–J). Additionally, SWCNTs-treated MLE-12 cells were largely positive for SA-GLB1 staining in time-dependent manner (Fig. 3K, L). The production of the profibrotic SASP factors PAI-1 and TGF-β were also markedly enhanced in the culture supernatants of SWCNTs-treated MLE-12 cells (Fig. 3M, N). Taken together, these results suggest that SWCNTs induce autophagic flux impairment and promote senescence with time-dependent SASP elevation in AECIIs.

**Fig. 4:**
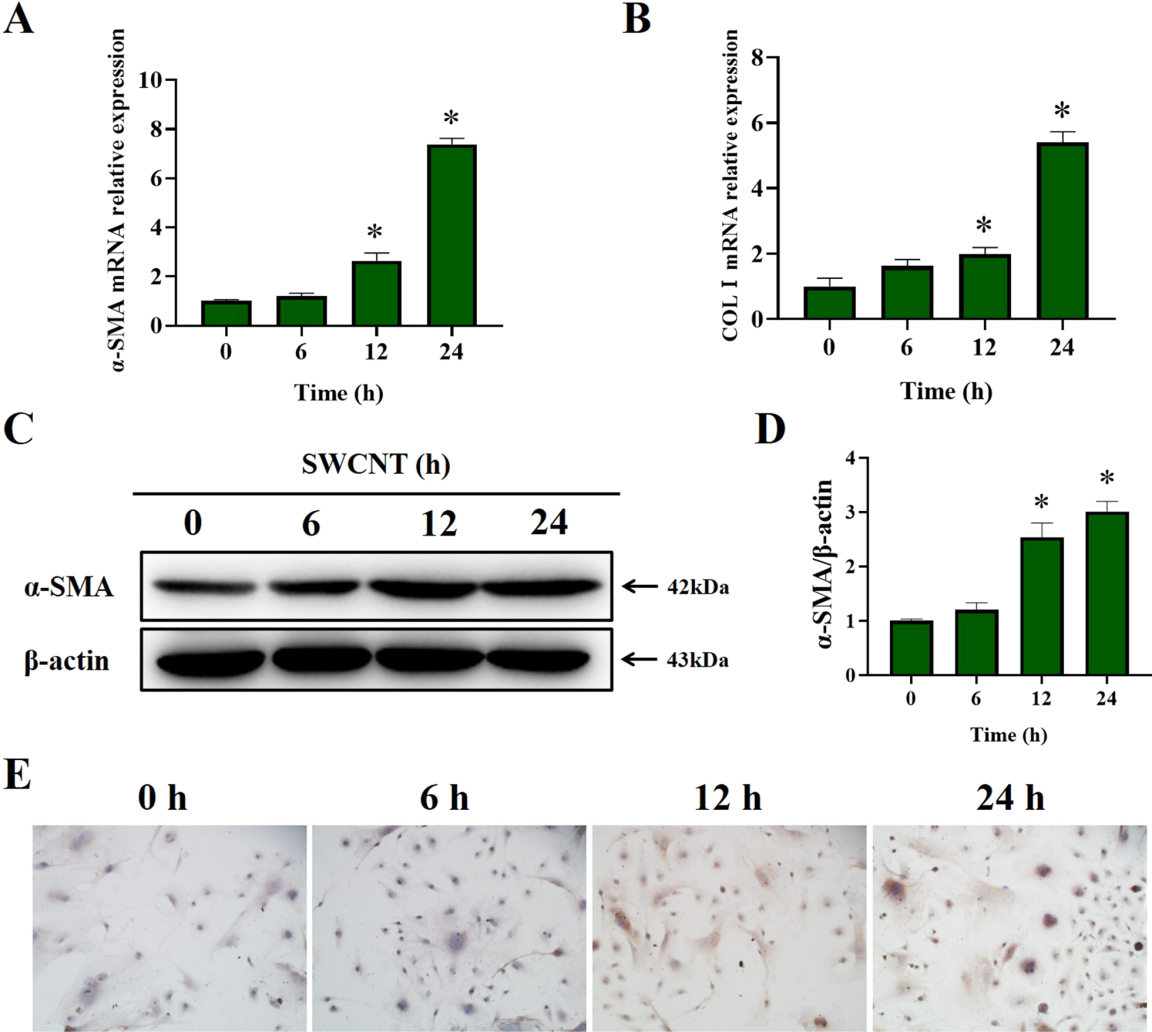
SWCNTs-exposed MLE-12 cells caused FMT. MLE-12 cells were treated with 10 μg/ml SWCNTs for 6, 12 and 24 h, and then cell-free culture medium, as conditioned medium (CM), was used to culture primary lung fibroblasts for 48 h. **A**, **B** mRNA expressions of α-SMA and COLI in lung fibroblasts were measured by qRT-PCR (n = 4). **C**, **D** Western Blotting analysis of α-SMA protein expression in lung fibroblasts (n = 3). **E** COLI positive expression in lung fibroblasts was evaluated by IHC (n = 5) (200×). *P < 0.05 vs CTRL group

Since we have observed impaired autophagic flux and senescence of AECIIs induced by SWCNTs, we carried out a CM experiment to address whether SWCNTs-induced impairment of autophagic flux and regulating senescence of AECIIs exacerbated pulmonary fibrosis. The supernatants of culture medium from PBS- and SWCNTs-treated MLE-12 cells were collected and used as CM to culture with primary lung fibroblasts. As shown in Fig. 4, there were no significant changes in α-SMA or COLI (myofibroblast markers) mRNA and protein expressions after treatment with CM from SWCNT-exposed MLE-12 cells for 6 h. However, after treatment with CM from SWCNT-exposed MLE-12 cells for 12 and 24 h, primary lung fibroblasts displayed increased mRNA and protein expressions of the myofibroblast markers a-SMA and COLI. Overall, these results indicate that SWCNTs-induced senescent MLE-12 cells trigger FMT and production of collagen via SASP secretion.

### Restoring autophagy alleviated SWCNTs-caused FMT through suppressing AECIIs senescence

**Fig. 5:**
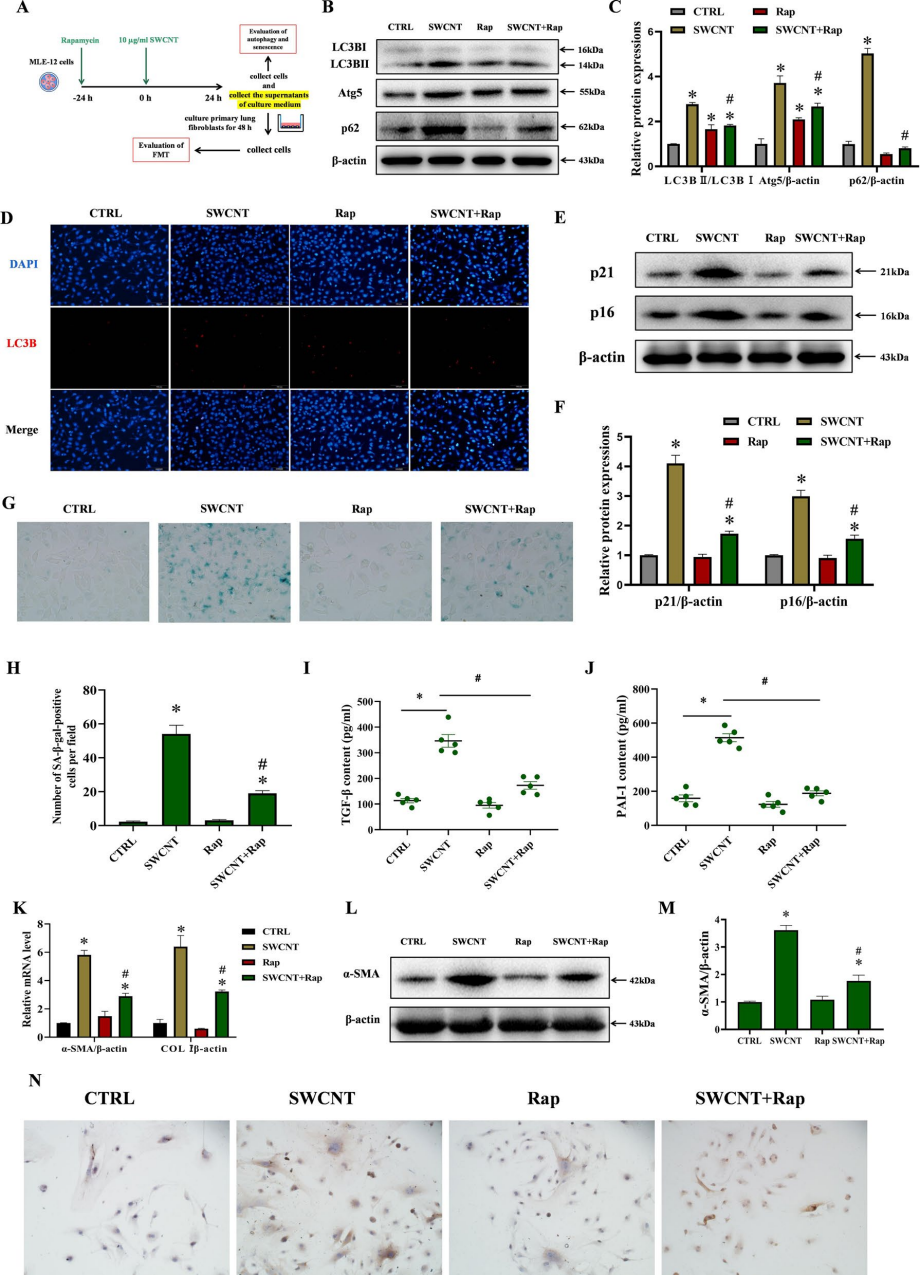
Restoration of autophagic flux using rapamycin alleviated FMT in lung fibroblasts by suppressing SWCNTs-triggered senescence in MLE-12 cells. MLE-12 cells were exposed to 10 μg/ml SWCNTs for 24 h with or without rapamycin pretreatment, and then the cell-free culture medium, as CM, was used to culture primary lung fibroblasts for 48 h. **A** Scheme of workflow for evaluating the effects of rapamycin on autophagy and senescence in MLE-12 cells and FMT in lung fibroblasts after SWCNTs exposure in vitro. **B**, **C** Western Blotting analysis of Atg5, LC3BI, LC3BII and p62 protein expressions in MLE-12 cells (n = 3). **D** LC3B puncta in MLE-12 cells was observed by IF (200×). **E**, **F** Western Blotting analysis of p21 and p16 protein expressions in MLE-12 cells (n = 3). **G** SA-β-gal activity was determined by X-gal staining in MLE-12 cells (400×). **H** The number of SA-β-gal positive MLE-12 cells per field (n = 8). Contents of TGF-β (**I**) and PAI-1 (**J**) in the supernatants of culture medium quantified by ELISA (n = 5). **K** mRNA expressions of α-SMA and COLI in lung fibroblasts were measured by qRT-PCR (n = 4). **L**, **M** Western Blotting analysis of α-SMA protein expression in lung fibroblasts (n = 3). **N** COLI positive expression in lung fibroblasts was evaluated by IHC (n = 5) (200×). *P < 0.05 vs CTRL group. ^#^*P* < 0.05 vs SWCNT group

Activation of autophagy was reported to be capable of protecting against cellular senescence and pulmonary fibrosis [35]. Therefore, we explored the effect of autophagy activation with rapamycin treatment on SWCNTs-induced senescence and FMT in MLE-12 cells (Fig. 5A). Rapamycin pretreatment reduced the up-regulated protein levels of Atg5, LC3B-II and p62 by SWCNTs, indicating that rapamycin partially restores SWCNTs-impaired autophagic flux (Fig. 5B, C). Moreover, IF results clearly indicated the SWCNTs-induced increase in LC3B puncta was also markedly decreased by rapamycin in MLE-12 cells, suggesting that rapamycin promotes the degradation of accumulated autophagosomes induced by SWCNTs (Fig. 5D). The administration of SWCNTs to MLE-12 cells elevated the protein expressions of p16 and p21, while pretreatment with rapamycin significantly reduced p16 and p21 protein expressions (Fig. 5E, F). SWCNT enhanced positive SA-GLB1 staining, but was significantly decreased by rapamycin pretreatment in MLE-12 cells (Fig. 5G, H). Additionally, rapamycin reduced SWCNTs-upregulated SASP factors (TGF-β and PAI-1) in the supernatants of culture medium (Fig. 5I, J). These data indicate that rapamycin decreases SWCNTs-mediated cellular senescence of AECIIs via the restoration of autophagic flux.

To elucidate whether rapamycin alleviates FMT in lung fibroblasts by suppressing SWCNTs-elicited senescent AECIIs, primary lung fibroblasts were incubated with CM for 48 h, then the FMT level was evaluated. The mRNA and protein expressions of α-SMA and COLI in the SWCNT group were significantly higher than those in the CTRL group (Fig. 5K–N). The differences in α-SMA and COLI mRNA and protein expressions between the Rap group and the CTRL group were not statistically significant (Fig. 5K–N). However, the mRNA and protein expressions of α-SMA and COLI in the SWCNT + Rap group were significantly lower than those in the SWCNT group (Fig. 5K–N). Together, these results indicate that restoration of autophagic flux leads to decreased cellular senescence in AECIIs, thereby alleviating SWCNTs-driven pulmonary fibrosis.

### Inhibiting autophagy aggravate SWCNTs-caused FMT through boosting AECIIs senescence

**Fig. 6:**
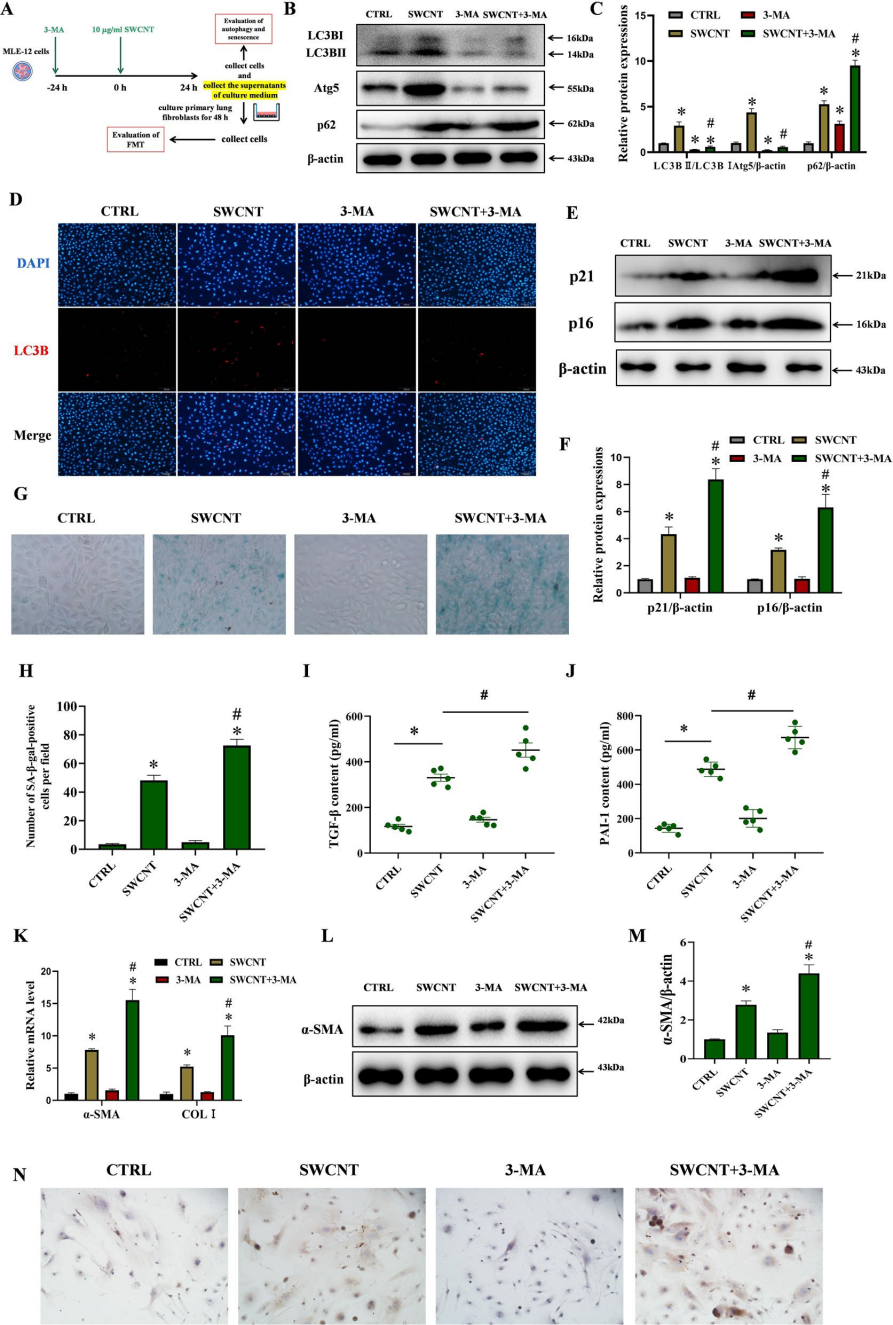
Inhibition of autophagy using 3-MA aggravated FMT in lung fibroblasts by promoting SWCNTs-triggered senescence in MLE-12 cells. MLE-12 cells were exposed to 10 μg/ml SWCNTs for 24 h with or without 3-MA pretreatment,, and then the cell-free culture medium, as CM, was used to culture primary lung fibroblasts for 48 h. **A** Scheme of workflow for evaluating the effects of 3-MA on autophagy and senescence in MLE-12 cells and FMT in lung fibroblasts after SWCNTs exposure in vitro. **B**, **C** Western Blotting analysis of Atg5, LC3BI, LC3BII and p62 protein expressions in MLE-12 cells (n = 3). **D** LC3B puncta in MLE-12 cells was observed by IF (200×). **E**, **F** Western Blotting analysis of p21 and p16 protein expressions in MLE-12 cells (n = 3). **G** SA-β-gal activity was determined by X-gal staining in MLE-12 cells (400×). **H** The number of SA-β-gal positive MLE-12 cells per field (n = 8). Contents of TGF-β (**I**) and PAI-1 (**J**) in the supernatants of culture medium quantified by ELISA (n = 5). **K** mRNA expressions of α-SMA and COLI in lung fibroblasts were measured by qRT-PCR (n = 4). **L**, **M** Western Blotting analysis of α-SMA protein expression in lung fibroblasts (n = 3). **N** COLI positive expression in lung fibroblasts was evaluated by IHC (n = 5) (200×). *P < 0.05 vs CTRL group. ^#^*P* < 0.05 vs SWCNT group

Finally, 3-MA, an autophagy inhibitor, was used to further verify the protective effect of autophagy on SWCNTs-induced cellular senescence and FMT (Fig. 6A). The autophagy was inhibited by 3-MA, as evidenced by decreased Atg5 expression and the ratio of LC3-II/LC3-I and increased p62 protein level (Fig. 6B, C). As shown in Fig. 6D, the SWCNTs-induced increase in LC3B puncta was also markedly decreased by 3-MA in MLE-12 cells. The determination of senescence in MLE-12 cells using the protein levels of p16 and p21 and positive SA-GLB1 staining revealed that 3-MA pretreatment aggravated SWCNTs-triggered cellular senescence (Fig. 6E–H). In consistent with the above data, the result on ELISA showed that 3-MA significantly increased SWCNT-upregulated SASP factors (TGF-β and PAI-1) in the supernatants of culture medium (Fig. 6I, J). These data demonstrate that 3-MA aggravates SWCNT-triggered cellular senescence, which is related to the inhibition of autophagy.

To confirm 3-MA aggravates FMT in lung fibroblasts by promoting SWCNT-induced senescence of AECIIs, CM experiments were conducted. As shown in Fig. 6K–N, no obvious changes of α-SMA and COL I mRNA and protein expressions were observed between the 3-MA group and the CTRL group. However, the mRNA and protein expressions of α-SMA and COLI in the SWCNT + 3-MA group were significantly higher than those in the SWCNT group (Fig. 6K–N). From these data, we can conclude that the aberrant exacerbation of senescence and pulmonary fibrosis caused by SWCNTs could be related to an accompanying autophagy blockage because the latter has been shown to contribute to pulmonary fibrosis by inducing senescence of AECIIs.

## Discussion

In this study, we found that autophagic flux impairment contributed to SWCNTs-induced senescence of AECIIs mediated pulmonary fibrosis in vivo and in vitro, and the restoration of autophagic flux using rapamycin could alleviate SWCNTs-mediated FMT by suppressing senescence of AECIIs and SASP factor secretion in vitro. In contrast, inhibition of autophagy using 3-MA aggravated SWCNTs-mediated FMT by promoting senescence of AECIIs and SASP factor secretion. To the best of our knowledge, this is the first study to demonstrate the causal link between autophagy and senescence during SWCNTs-induced pulmonary fibrosis. These findings provide novel evidence that the restoration of autophagic flux is a promising strategy to counteract senescence, which will hopefully contribute to the development of new strategies against CNTs-induced pulmonary fibrosis.

The concentration of CNTs differs in various occupational environment. Erdely et al. found that an inhalable elemental carbon mass concentration arithmetic mean of 10.6 μg/m^3^ was found among workers exposed to CNTs [3]. One previous study reported that a dose of 80 μg/mouse was equal to a predicted human lung burden based on the equivalent alveolar surface area for a person performing light work at 7 μg/m^3^ for 13 years [32]. A single dose of 40 μg SWCNTs was administered by intratracheal instillation in our study, which is a common route of administration to deliver a specific dose of respirable materials, such as CNTs, into animal lungs. The exposure dosage in the current study exceeded REL of 1 μg/m^3^ CNT. However, this exposure dosage, which has been shown to produce sufficient lung burdens, approached environmental exposure concentration in workplace [32]. Thus, it was suitable to conduct this research using the dose of 40 μg/mouse in vivo.

Cellular senescence is a state of irreversible growth arrest and is implicated in the development of pulmonary fibrosis [36–38]. Numerous studies have shown that SASP factors, which are a broad repertoire of cytokines, chemokines, matrix remodelling proteases and growth factors, are secreted by senescent cells and are involved in the pathophysiological progression of pulmonary fibrosis [39, 40]. The clearance of senescent cells has been shown to improve pulmonary function and inhibit the fibrotic response in animal models [41, 42]. SASP-related TGF-β and PAI-1 are recognized as the pivotal inducers of pulmonary fibrosis [10, 43] Lucas et al. revealed that MWCNTs exacerbated PAI-1 protein expression in TGF-β-exposed BEAS-2B cells [43]. Our previous study confirmed that TGF-β contributed to CNTs-mediated FMT in vitro [10]. There is insufficient evidence on the role of senescence in SWCNTs-exposed AECIIs and subsequent pulmonary fibrosis. Our investigations indicated that SWCNTs induced senescence with elevated profibrotic SASP factors TGF-β and PAI-1 in vivo and in vitro. To further verify whether cellular senescence mediates SWCNTs-induced pulmonary fibrosis, we performed CM experiments to examine the causative association of SWCNTs-induced senescence in AECIIs and FMT in lung fibroblasts. The results showed that exposure of MLE-12 cells to SWCNTs for 24 h induced FMT in lung fibroblasts, suggesting that SWCNTs-triggered senescence in AECIIs mediates pulmonary fibrosis.

Autophagy is an essential catabolic mechanism for the degradation of cellular components, long-lived cytosolic proteins and damaged organelles in response to a variety of stimuli. During autophagy, portions of the cytosol or organelles are sequestered by double-membrane autophagosomes, which fuse with lysosomes and are degraded by resident hydrolases [44]. The dysregulation of autophagy is linked to a wide range of pathologic conditions induced by diseases, ageing and toxic substances. The effect of autophagy on the toxicity of chemicals is not always consistent but may vary depending on cellular context. Suppression of autophagy promoted FMT by decreasing apoptosis in fibroblasts during pulmonary fibrosis [45]. Impaired autophagy significantly exacerbated inflammatory response and induced abundant collagen accumulation [46]. However, Ghavami et al. reported that excessive autophagy promoted pulmonary fibrosis through the excessive biosynthesis of collagen and fibronectin [47]. Mittal et al. found that autophagic flux disruption mediated carbon nanofiber-induced apoptosis in human lung epithelial cells through oxidative stress [48]. Whether autophagy acts positively or negatively on cellular senescence and fibrosis is still unclear. AECIIs are a metabolically active lung cell population that is important for surfactant biosynthesis and alveolar homeostasis, have emerged as a key player in the development of pulmonary fibrosis [16]. To date, a few studies have been published regarding the effects of CNTs-induced autophagy in AECIIs on pulmonary fibrosis. The current study found that SWCNTs markedly upregulated the protein expressions of Atg5, p62 and LC3B-II, which are three specific autophagy markers, in AECIIs in vivo and vitro. It is possible that the upregulated LC3B-II, Atg5 and p62 in SWCNTs-exposed lungs of mice and MLE-12 cells could reflect the impairment of autophagic flux.

Studies have demonstrated that autophagic flux impairment was closely involved in senescence, and senescent cell-associated profibrotic factors were linked to myofibroblast activation [35, 49, 50]. Rapamycin, which is an autophagy inducer, facilitates the clearance of abnormal proteins or mitochondria by autophagy and makes cells more resistant to toxic stimulation [51, 52]. The protective effect of rapamycin can also be attributed to the regulation of senescence and SASP factors, such as TGF-β and PAI-1 [53, 54]. Therefore, we pretreated MLE-12 cells with rapamycin, and then CM experiments were conducted to clarify the role of SWCNTs-impaired autophagic flux in senescence in AECIIs and subsequent pulmonary fibrosis. The current study showed that rapamycin restored SWCNT-impaired autophagic flux in MLE-12 cells and further alleviated SWCNT-induced senescence in MLE-12 cells. Additional CM experiments confirmed that rapamycin inhibited FMT induced by SWCNT-exposed senescent AECIIs, which was related to the restoration of autophagic flux. To further verify the aforementioned findings, autophagy in SWCNTs-exposed MLE-12 cells was inhibited using 3-MA, then senescence in MLE-12 cells and FMT were evaluated. We found that autophagy inhibition in AECIIs aggravated SWCNTs-induced senescence and subsequent FMT. These data further verify the protective effect of autophagy on SWCNTs-triggered senescence of AECIIs mediated FMT. However, it is unknown whether restoring autophagic flux ameliorates SWCNTs-induced pulmonary fibrosis in vivo and we need to further study in the future.

## Conclusion

In summary, we demonstrated that SWCNTs triggered senescence of AECIIs by impairing autophagic flux mediated pulmonary fibrosis. The findings raise the possibility of using senescence-related cytokines as potential biomarkers for hazard of CNTs exposure, and regulating autophagy as a promising target to halt CNT-induced fibrosis development.

## Supplementary Information

**Additional file 1: Table S1.** Gene-specific primers.

## Data Availability

The datasets used and/or analysed during the current study are available from the corresponding author on reasonable request.

## Abbreviations

AECIs: Alveolar type I epithelial cells
AECIIs: Alveolar type II epithelial cells
Atg5: Autophagy related 5
BALF: Bronchoalveolar lavage fluid
CM: Conditioned medium
CNTs: Carbon nanotubes
COVID-19: Coronavirus disease 2019
DMEM: Dulbecco’s modified Eagle’s medium
EMT: Epithelial–mesenchymal transition
FBS: Foetal bovine serum
FMT: Fibroblast-myofibroblast transdifferentiation
HE: Haematoxylin and eosin
HYP: Hydroxyproline
IF: Immunofluorescence
IHC: Immunohistochemistry
MTT: 3-(4,5)-Dimethylthiahiazo(-z-y1)-3,5-di-phenytetrazoliumromide
MWCNTs: Multi-walled CNTs
PAI-1: Plasminogen activator inhibitor-1
RT–qPCR: Real-time quantitative polymerase chain reaction
SA-β-Gal: Senescence-associated-β-galactosidase
SASP: Senescence-associated secretory phenotype
SWCNTs: Single-walled CNTs
TGF-β: Transforming growth factor-β
