# Supplementary material for: Impaired autophagy-accelerated senescence of alveolar type II epithelial cells drives pulmonary fibrosis induced by single-walled carbon nanotubes
Source: J Nanobiotechnology. 2023 Feb 28;21:69. doi: 10.1186/s12951-023-01821-6 (PMC9970859; doi:10.1186/s12951-023-01821-6)
Supplement: Supplementary file 1 — Additional file 1: Table S1. Gene-specific primers. [file 12951_2023_1821_MOESM1_ESM.docx]

**Table S1 Gene-specific primers**

| **Gene** | **Forward primer** | **Reverse primer** |
| --- | --- | --- |
| α-SMA | GTACCACCATGTACCCAGGC | GCTGGAAGGTAGACAGCGAA |
| CollagenⅠ | TTCTCCTGGCAAAGACGGAC | CGGCCACCATCTTGAGACTT |
| β-actin | CGCCCTAGGCACCAGGGTGTG | TCGGTGAGCAGCACAGGGTG |
